# Interaction of Copper Trafficking Proteins with the Platinum Anticancer Drug Kiteplatin

**DOI:** 10.1002/cmdc.202100593

**Published:** 2021-11-15

**Authors:** Alessandra Barbanente, Angela Galliani, Rosa Maria Iacobazzi, Alessia Lasorsa, Maria Incoronata Nardella, Antonio Pennetta, Nicola Margiotta, Fabio Arnesano

**Affiliations:** ^1^ Department of Chemistry University of Bari “Aldo Moro” Via E. Orabona 4 70125 Bari Italy; ^2^ Laboratory of Experimental Pharmacology IRCCS Istituto Tumori “Giovanni Paolo II” Viale O. Flacco 65 70124 Bari Italy; ^3^ Department of Engineering for Innovation University of Salento Via per Monteroni Km 1 73100 Lecce Italy; ^4^ Department of Cultural Heritage University of Salento Via Dalmazio Birago 64 73100 Lecce Italy

**Keywords:** Platinum, Antitumor agents, Copper, Mass spectrometry, Circular dichroism

## Abstract

The interaction of metallodrugs with proteins influences their mechanism of action and side effects. In the case of platinum drugs, copper transporters modulate sensitivity and resistance to these anticancer agents. To deepen the knowledge of the structural properties underlying the reactivity of platinum drugs with copper transporters, we studied the interaction of kiteplatin and two of its derivatives with the methionine‐rich motif of copper importer Ctr1 and with the dithiol motif of the first domain of Menkes ATPase. Furthermore, cellular uptake and cytotoxicity of the three complexes were evaluated in cisplatin‐sensitive and ‐resistant ovarian cancer cells, comparing the data with those of clinically relevant drugs. Reactivity depends on the tightness of the chelate ring formed by the carrier ligands and the nature of the leaving and entering groups. The results highlight the importance of subtle changes in the platinum coordination sphere that affect drug absorption and intracellular fate.

## Graphical abstract

**Kiteplatin, a promising anticancer drug** that overcomes resistance to cisplatin, binds to a methionine motif of Cu importer Ctr1 and to a dithiol motif of the first domain of Menkes ATPase, which can modulate drug uptake and intracellular distribution. The interaction is influenced by the type of incoming ligands, the nature of the leaving groups (two chlorido ligands) and the chelating character of the carrier ligand (1,4‐diaminocyclohexane).

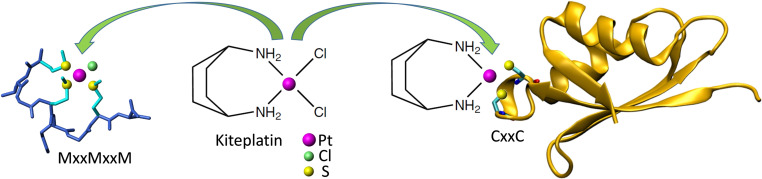

## Introduction

The discovery of cisplatin has revolutionized the treatment of certain types of tumors, like ovarian, testicular and lung cancer.[^1^, ^2^] The cytotoxicity of this antitumor drug is mediated by its interaction with DNA to form adducts that can result in the generation of an apoptotic signal.^[3]^ However, DNA damage can be attenuated and the resistance that follows is a major limitation of cisplatin‐based chemotherapy.^[4]^

**Scheme 1:**
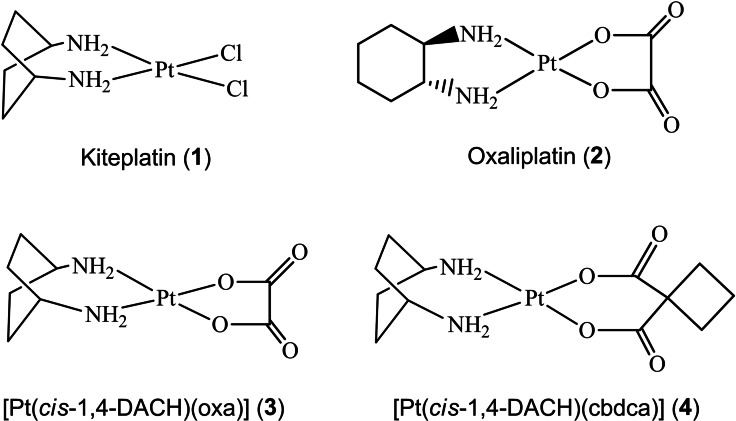
Platinum complexes used in this investigation.

For this reason, one of the main goals of current anticancer research is the synthesis of new platinum (Pt) drugs capable to overcome cisplatin resistance either acquired during cycles of therapy or intrinsic (for example in patients with colorectal, prostate, lung or breast cancer).^[5]^ Therefore, new generation Pt complexes such as oxaliplatin, that contains a 1*R*,2*R*‐diaminocyclohexane (1*R*,2*R*‐DACH) as carrier ligand, and kiteplatin, [PtCl_2_(*cis*‐1,4‐DACH)] (**1** in Scheme 1), with an isomeric form of oxaliplatin diamine ligand, were designed.^[6]^

Experimental evidences support the role of copper (Cu) transporters in the uptake of and resistance to cisplatin and its derivatives, confirming the tight connection between Cu homeostasis and cellular response to Pt‐drugs.[^7^, ^8^, ^9^]

The high‐affinity Cu influx transporter Ctr1 transfers Cu(I) ions across the plasma membrane to distinct cytosolic soluble chaperones, which ultimately supply Cu to cuproenzymes.[^10^, ^11^, ^12^, ^13^, ^14^]

Human Ctr1 (hCtr1) is a glycosylated protein of 190 amino acids, which consists of three putative transmembrane (TM) segments,^[15]^ an extracellular N‐terminal domain, which is rich in methionine (Met) residues that are essential for high affinity Cu uptake,^[16]^ and an intracellular C‐terminal domain. When the Ctr1 gene was deleted or silenced, increased resistance to cisplatin occurred which was correlated with reduced drug uptake and reduced cell‐death caused by Pt‐DNA adducts.[^8^, ^17^, ^18^]

In addition to the downregulation of Ctr1, an upregulation of two Cu pumps, ATP7A and ATP7B, may also play a key role in resistance to Pt‐based drugs.[^19^, ^20^, ^21^]

ATP7A is a Cu‐exporting P‐type ATPase which regulates the cytoplasmic Cu concentration; in particular, it is involved in the transport of Cu(I) through the secretory pathway and in Cu(I) excretion across the plasma membrane.^[22]^ Previous studies have shown that ATP7A silencing increases sensitivity to cisplatin treatment in various types of solid tumors, whereas forced expression of the protein increases drug resistance,[^23^, ^24^] possibly because ATP7A can confine cisplatin in subcellular compartments (such as lysosomes).^[25]^

We demonstrated that, when the cytoplasmic *N*‐terminal domain of ATP7A (Mnk1, hereafter) was incubated with cisplatin or oxaliplatin, the protein was able to bind Pt(II) in a bidentate manner, with the two‐cysteine (Cys) motif CxxC replacing the chlorido ligands and the oxalate group, respectively.[^26^, ^27^] We also showed that the octapeptide MTGMKGMS (called Mets7), featuring a Met‐rich motif of yeast Ctr1, caused the complete loss of cisplatin ligands and coordinated the Pt(II) ion in a tetradentate manner, switching from a random coil to a β‐turn conformation.[^28^, ^29^] Similar results were subsequently obtained using homologous Met‐rich motifs of human Ctr1.[^30^, ^31^] In contrast, when the reaction was performed with transplatin, the clinically ineffective *trans* isomer of cisplatin, no release of ammine ligands was observed.^[28]^ Since the ammine ligands are required for the antitumor activity of cisplatin, we proposed that, after the initial interaction with Met‐rich motifs of Ctr1, an endocytic process is induced which incorporates a portion of extracellular medium containing intact cisplatin molecules into vesicles that are delivered to subcellular compartments, including the nucleus.[^19^, ^28^, ^32^]

Kiteplatin has better antitumor activity than cisplatin in most cisplatin‐resistant cancer cell lines and is also active in some oxaliplatin‐resistant cells (such as LoVo‐OXP).^[33]^ *In vivo* experiments have also shown that this compound possesses significant activity against cisplatin‐resistant murine leukemias.^[33]^ For these reasons, kiteplatin has been extensively investigated as a potential new Pt anticancer drug.

In this study, we extended our previous investigations of kiteplatin's mechanism of action by measuring drug uptake and cytotoxicity in cisplatin‐sensitive and ‐resistant ovarian cancer cell lines. To assess whether kiteplatin's ability to circumvent resistance is related to the interaction with proteins involved in Cu trafficking, we tested whether Mets7 and Mnk1 bind kiteplatin and its derivatives [Pt(*cis*‐1,4‐DACH)(oxa)] (oxa=oxalate; **3** in Scheme 1) and [Pt(*cis*‐1,4‐DACH)(cbdca)] (cbdca=1,1‐cyclobutanedicarboxylate; **4** in Scheme 1). Moreover, since these complexes contain an isomeric form of the oxaliplatin diamine ligand, we compared their interaction with Mets7 and Mnk1 with that of oxaliplatin [Pt(1*R*,2*R*‐DACH)(oxa)] (**2** in Scheme 1), carrying out investigations with electrospray ionization mass spectrometry (ESI‐MS) and circular dichroism (CD).

## Results and Discussion

The *in vitro* cytotoxicity of kiteplatin (complex **1**) was formerly tested in the pair of ovarian cancer cell lines A2780/A2780cisR (sensitive/resistant to cisplatin) where the complex showed IC_50_ values (MTT test; 72 h treatment) of 0.8 and 4.0 μM, respectively, resulting more potent than cisplatin, which showed IC_50_ values equal to 2.7 and 21.6 μM, respectively. Moreover, kiteplatin was able to partially overcome cisplatin resistance, as evidenced by the resistance factor (RF, defined as IC_50_ resistant/parent line) of 5.0 to be compared with that of cisplatin (RF=8.0). Since Pt accumulation is critical for the cytotoxicity of a Pt drug, cellular accumulation of kiteplatin in the A2780 cell line was evaluated by measuring the Pt content after 4 h (4.7 pmol Pt/10^6^ cells) and 24 h (38 pmol Pt/10^6^ cells), resulting ca. 1.3–2.0 higher than that of cisplatin.^[34]^

Kiteplatin was also tested (MTT test; 72 h treatment) on a panel of human tumor cell lines, including cervical (A431) and breast (MCF‐7) cancer along with a melanoma (A375) and four different colon cancer cell lines (HCT‐15, SW480, CaCo‐2, DLD‐1) corresponding to different stages of tumor progression. Complex **1** was, on average, slightly more effective than oxaliplatin and much more effective than cisplatin. In particular, it resulted more effective than cisplatin by a factor of 1.1–5.7. In addition, complex **1** was able to overcome resistance to cisplatin while complexes **3** and **4** were not.[^33^, ^35^]

Since the involvement of Ctr1 in cisplatin sensitivity/resistance in the well characterized A2780/A2780cis human ovarian carcinoma cell line pair has been well documented,^[36]^ we decided to focus on our in‐house available A2780/A2780/Cp8 cell line pair and carried out an *in vitro* investigation on compounds **1**, **3**, and **4** using cisplatin and oxaliplatin as reference compounds. The cytotoxicity was evaluated by means of the MTT test after 72 h treatment with increasing concentrations of the tested compounds up to 50 μM. Dose‐survival curves were used to calculate the IC_50_ values reported in Table 1.

**Table 1:** *In vitro* cell viability.^[a]^

	IC_50_ [μM]±S.D.		
Compounds	A2780	A2780/Cp8	RF
Kiteplatin (**1**)	0.54±0.05	1.7±0.2	3.1
[Pt(*cis*‐1,4‐DACH)(oxa)] (**3**)	2±0.3	5.1±0.9	2.6
[Pt(*cis*‐1,4‐DACH)(cbdca)] (**4**)	30±6	>50	–
Cisplatin	8±1	22±5	2.8
Oxaliplatin (**2**)	2.2±0.2	4.5±1.1	2.0

[a] Cells (*ca*. 5000 cells per well) were treated for 72 h with increasing concentrations (in the range 0.125–50 μM) of the tested compounds. Cytotoxicity was assessed by the MTT test. IC_50_ values were calculated using nonlinear regression. S.D.=standard deviation. Resistance Factor (RF)=IC_50_(resistant)/IC_50_(parent line).

In the cisplatin‐sensitive A2780 cell line, with the exception of compound **4** (having the cbdca dicarboxylate leaving group present in carboplatin), all the compounds resulted to be superior to cisplatin. Kiteplatin (**1**) confirmed to be more active than cisplatin but demonstrated also to possess an activity (IC_50_=0.54 μM) superior to that of oxaliplatin (IC_50_=2.2. μM). Among the four complexes containing DACH, **1** resulted the most active because of the presence of the two chlorido ligands. This complex, besides showing the highest uptake (see discussion below), once in the cell, is supposed to be hydrolyzed faster than the bis‐chelated dicarboxylato ligands (cbdca and oxa) present in compounds **2–4**. The two oxalato derivatives **2** and **3** appear to have a comparable activity in the A2780 cell line. To investigate the ability of the complexes to overcome the acquired resistance of human tumors we selected the A2780/Cp8 cell line, which is known for its resistance to cisplatin. Some of the main molecular mechanisms involved in cisplatin resistance of cancer cells have been identified in reduced drug uptake, high cellular glutathione and thioredoxin reductase levels, and in enhanced repair of DNA damage. Cross‐resistance profiles were evaluated by means of the RF, which is defined as the ratio between the IC_50_ value obtained for the resistant cell line and that obtained for the sensitive cell line (see Table 1). Although the RF for the four DACH complexes resulted to be comparable to that of cisplatin, indicating a partial cross‐resistance to the clinical drug, **1** appeared to be the most active compound also in the A2780/Cp8 cell line, with an IC_50_ value of 1.7 μM, *ca*. 13 times lower than that found for cisplatin and *ca*. 2.6 times lower than that of oxaliplatin (Table 1).

**Figure 1:**
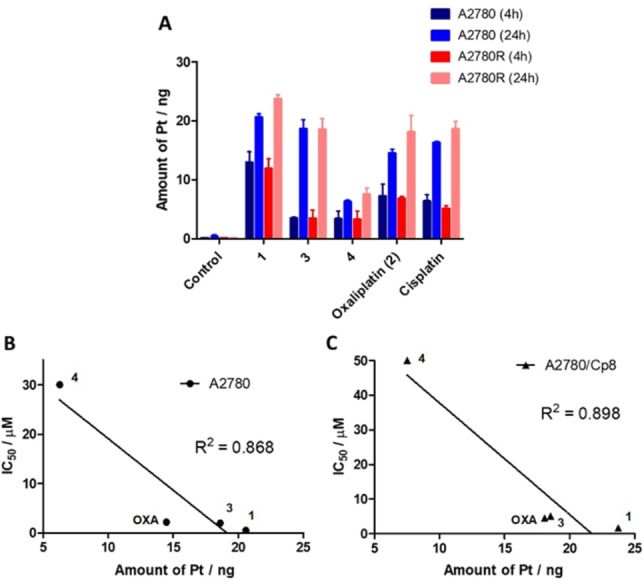
Cellular uptake of Pt compounds (A) and correlation between cytotoxicity and cellular Pt levels in drug‐treated A2780 (B) and A2780/Cp8 (C) cells. OXA=oxaliplatin (**2**).

Cellular uptake is an important factor influencing drug efficacy and in controlling resistance to Pt‐based drugs. Hence, uptake experiments were performed in the A2780/A2780/Cp8 human ovarian carcinoma cell line pair sensitive and resistant to cisplatin. Cancer cells were treated for 4 h and 24 h with equal concentration of the Pt compounds (10 μM), the intracellular Pt content was quantified by ICP‐MS and the results were summarized in Figure 1A. The Pt content was time dependent for all Pt complexes in both sensitive and resistant A2780 cells. However, the Pt levels after 4 h incubation resulted quite similar in both sensitive and resistant cells for all compounds. Interestingly, with the exception of compound **3**, the intracellular Pt content after 24 h treatment resulted to be higher in the resistant tumor cell line A2780/Cp8, indicating that mechanisms different from Pt influx or efflux might play a role in determining the reduced activity. The highest Pt content at both incubation times and in both tumor cell lines was found for kiteplatin (**1**; Figure 1A). Cisplatin, oxaliplatin and compound **3** showed similar uptake in both cell lines, while compound **4**, which was the least cytotoxic in both cell lines, resulted to be also the least accumulated Pt compound.

Finally, a rough direct and linear correlation was found (R^2^=0.868; Figure 1B) between the cytotoxic activity and cellular uptake of the four Pt−DACH compounds in the cisplatin‐sensitive A2780 cell line, while this correlation was better (R^2^=0.898; Figure 1C) in the case of the resistant A2780/Cp8 cell line.

In order to understand if the proteins involved in copper trafficking could be responsible for the different cytotoxicity of kiteplatin and its derivatives (also with reference to the ability to overcome cisplatin resistance), we decided to investigate the reaction with Mets7, a model peptide of Cu(I) importer Ctr1, and with Mnk1, the first soluble domain of Cu(I) efflux pump ATP7A.

**Figure 2:**
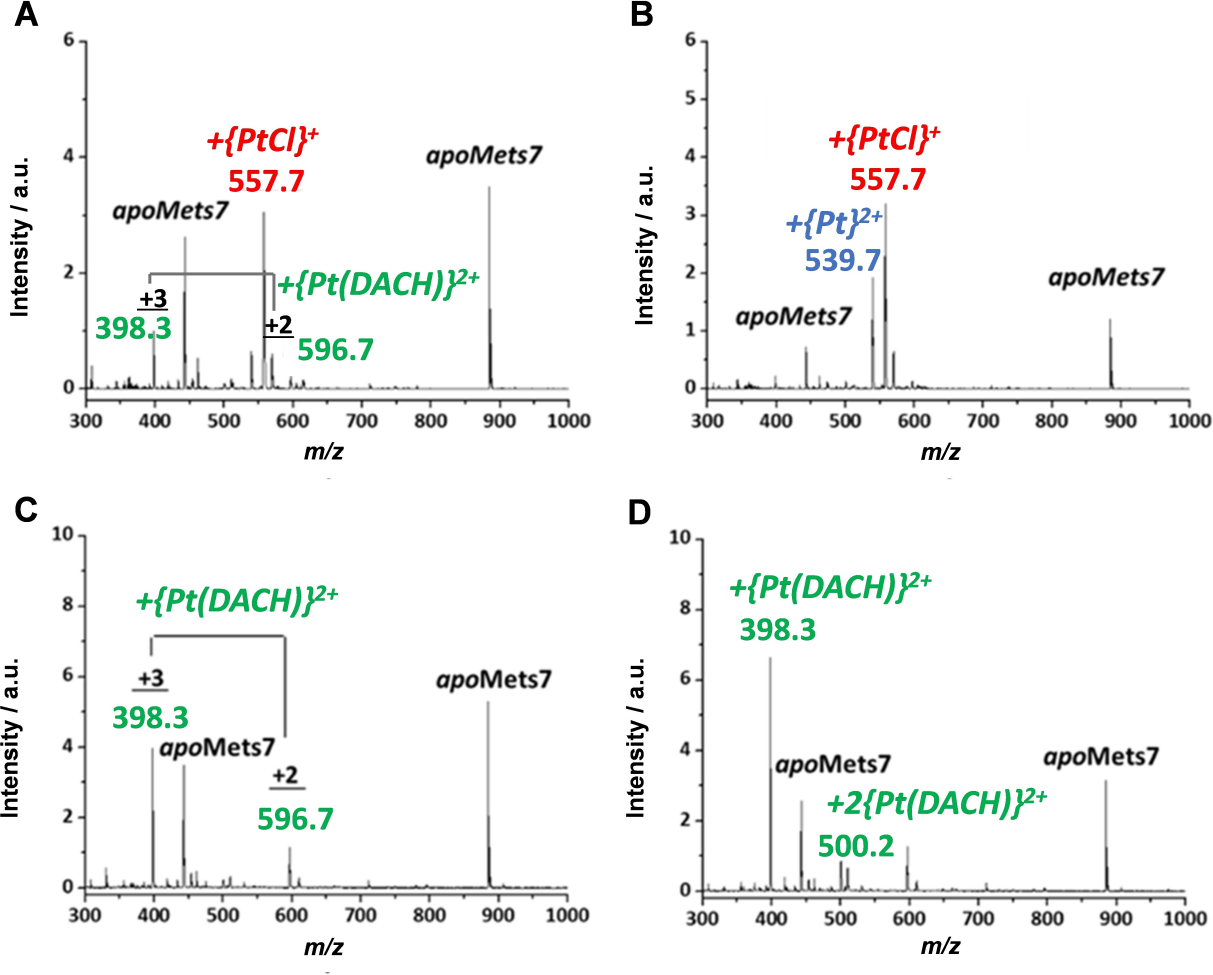
ESI‐MS spectra of Mets7 incubated with an equimolar amount of kiteplatin (**1**) (A and B) and oxaliplatin (**2**) (C and D), recorded after 24 h (A and C) and 48 h of incubation (B and D).

A solution of Mets7 was incubated with an equimolar amount of kiteplatin or oxaliplatin (50 μM final concentration) at 25 °C and the reaction course monitored by ESI‐MS (see Table 2). In the case of kiteplatin (**1**), soon after mixing, the spectrum recorded in positive ion mode showed two peaks corresponding to the *apo*peptide at *m/z* 884.5 and 442.8 (doubly charged). After 24 h, it was possible to appreciate a doubly charged peak at *m/z* 596.7, consistent with the binding of {Pt(*cis*‐1,4‐DACH)}^2+^ moiety to the peptide, and a stronger peak at *m/z* 557.7, which corresponded to the doubly charged species [Mets7+PtCl+H]^2+^ (Figure 2A). This latter species was the predominant adduct in solution after 48 h together with [Mets7+Pt]^2+^ (*m/z* 539.7) (Figure 2B).

**Table 2:** Assignment of adducts of Mets7 incubated with kiteplatin (**1**), oxaliplatin (**2**) and [Pt(*cis*‐1,4‐DACH)(oxa)] (**3**) observed by ESI‐MS.

Adduct assignment	Experimental *m/z* ^[a]^	Calculated *m/z* ^[a]^
*apo*Mets7		
[Mets7+H]^+^	884.5	884.4
[Mets7+2H]^2+^	442.8	442.7
Mets7+**1**		
[Mets7+Pt(1,4‐DACH)]^2+^	596.7	596.7
[Mets7+Pt(1,4‐DACH)+H]^3+^	598.3	398.1
[Mets7+PtCl+H]^2+^	557.7	557.6
[Mets7+Pt]^2+^	539.7	539.7
Mets7+**2**		
[Mets7+Pt(1*R*,2*R*‐DACH)]^2+^	596.7	596.7
[Mets7+Pt(1*R*,2*R*‐DACH)+H]^3+^	398.3	398.1
[Mets7+Pt(1*R*,2*R*‐DACH)+ Pt(1*R*,2*R*‐DACH)‐H]^3+^	500.2	500.2
Mets7+**3**		
[Mets7+Pt]^2+^	539.7	539.7

[a] The reported *m/z* ratios are referred to the prevailing isotopologue in the isotope pattern.

The reactivity of kiteplatin toward Mets7 is similar to that observed for cisplatin, i. e. the Pt(II) ion loses the carrier ligands (1,4‐DACH in kiteplatin and the two ammines in cisplatin) and one or two chlorido ligands.^[28]^

When oxaliplatin (**2**) was incubated with Mets7, the ESI‐MS spectrum recorded after 24 h of incubation showed the binding of {Pt(1*R*,2*R*‐DACH)}^2+^ to the peptide (Figure 2C). This predominant adduct was present as both doubly (*m/z* 596.7) and triply charged species (*m/z* 398.3). The intensity of these peaks increased after 48 h of incubation with the concomitant decrease in intensity of the *apo*peptide peak. Moreover, the spectrum showed a low intense peak at *m/z* 500.2 corresponding to triply charged species containing two {Pt(1*R*,2*R*‐DACH)}^2+^ moieties bound to the peptide (Figure 2D).

Summarizing this part, the ESI‐MS spectra indicate that, while the 1,4‐DACH ligand of kiteplatin is displaced by Mets7, 1*R*,2*R*‐DACH of oxaliplatin remains firmly bound to Pt(II).

**Figure 3:**
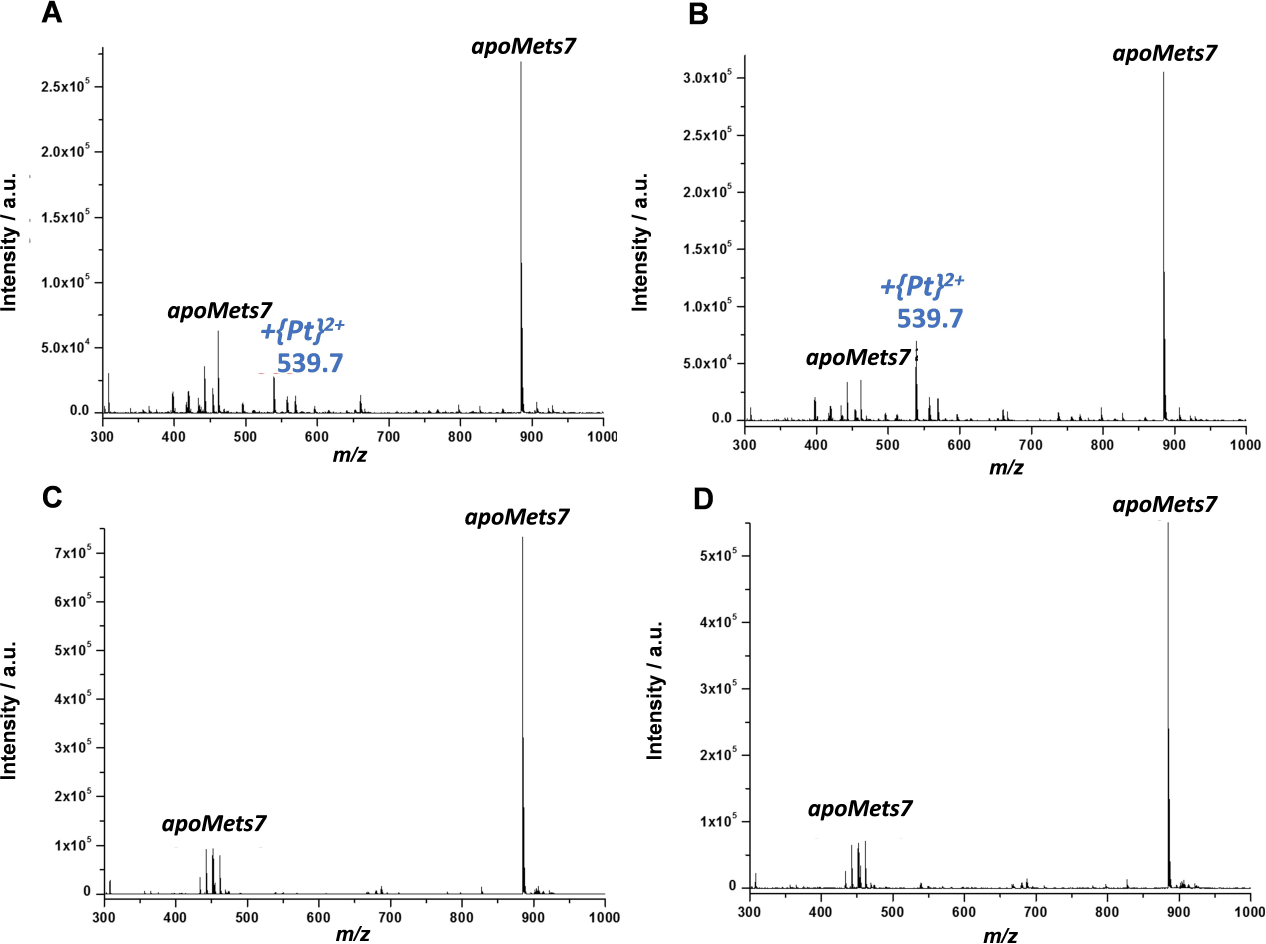
ESI‐MS spectra of Mets7 incubated with an equimolar amount of [Pt(*cis*‐1,4‐DACH)(oxa)] (**3**) (A and B) and [Pt(*cis*‐1,4‐DACH)(cbdca)] (**4**) (C and D), recorded after 24 h (A and C) and 48 h of incubation (B and D).

The peptide was also incubated with two derivatives of kiteplatin, the first having an oxalate (oxa) leaving group ([Pt(*cis*‐1,4‐DACH)(oxa)]) (**3**) and the second having a cyclobutanedicarboxylate (cbdca) leaving group ([Pt(*cis*‐1,4‐DACH)(cbdca)]) (**4**). The ESI‐MS spectrum obtained after 24 h of incubation of Mets7 with **3** showed that the predominant peak was that of the *apo*peptide *m/z* 884.5 (Figure 3A). In addition, a signal at *m/z* 539.7 was visible in the spectrum. The latter was assigned to [Mets7+Pt]^2+^ and became more intense after 48 h (Figure 3B). Analogously to the case of kiteplatin, Mets7 was able to displace 1,4‐DACH and oxa ligands from complex **3**, even though the *apo*peptide peak remained the most abundant signal, indicating a much slower reaction of Pt(II) with Mets when the chelating oxa ligand was present as a leaving group instead of two chlorido ligands.

Oppositely to the case of kiteplatin and complex **3**, there was no significant reaction between **4** (the cbdca derivative) and Mets7 after 48 h of reaction (Figure 3C and 3D). This means that complex **4** was able to retain its ligands. This low reactivity could be due to the steric hindrance of the cbdca leaving ligand compared to chlorido and the oxa ligands in kiteplatin and complex **3**, respectively. In fact, the crystal structure of **4** shows that the cyclobutane ring makes almost a right angle with the Pt coordination plane and could shield the metal from attack by an incoming nucleophile.^[37]^ Low reactivity with Mets7 was also found for carboplatin (which also contains a cbdca leaving group).^[38]^

**Figure 4:**
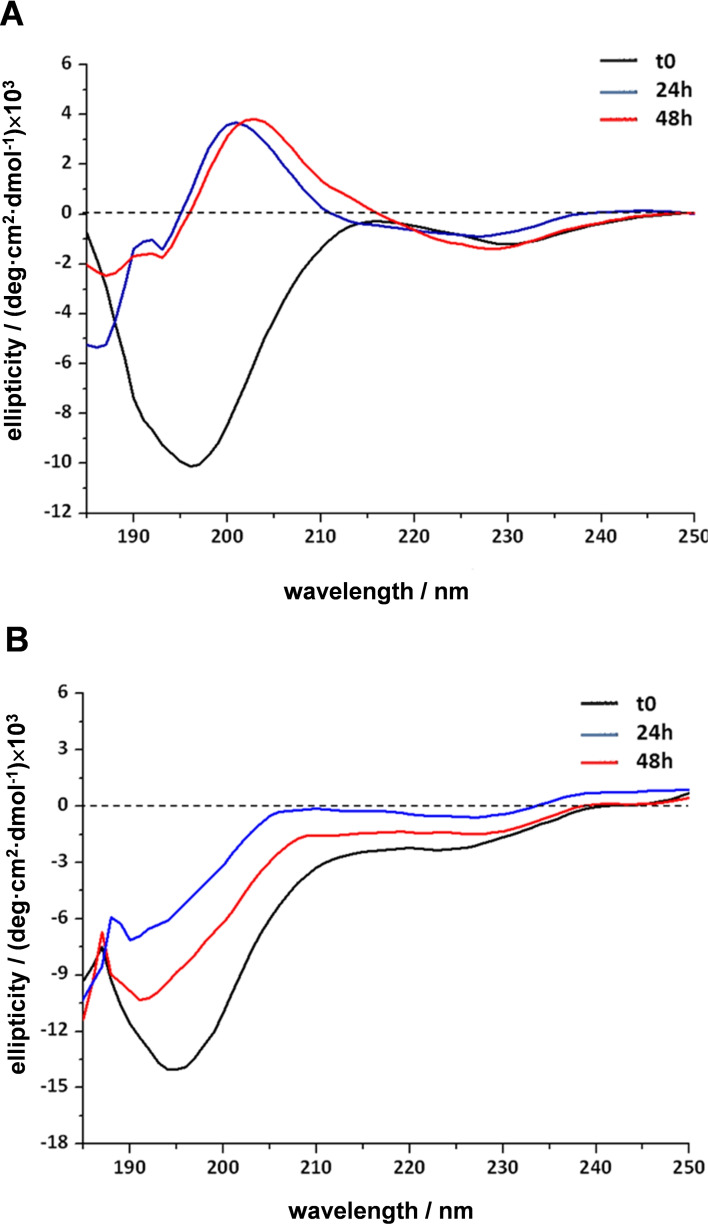
Far‐UV circular dichroism spectra of Mets7 incubated with one equivalent of kiteplatin (**1**) (A) and oxaliplatin (**2**) (B) recorded at different time intervals.

To obtain information on the peptide conformation induced by chelation to Pt, CD spectroscopy was used. This technique uses circularly polarized light to study structural aspects of chiral species. Indeed, it is mainly applied to study biological molecules such as proteins and peptides, their structure and interactions with metal ions and other molecules. In the far‐UV region of CD spectrum, free Mets7 (dissolved in MilliQ water) displayed a negative band at 195 nm, which is characteristic of an unstructured peptide. Addition of one equivalent of kiteplatin produced a conformational change after 24 h of incubation with the appearance of a positive band at 200 nm, corresponding to a β‐turn‐type structure (Figure 4A). After 48 h of incubation, when most of the peptide had reacted with kiteplatin to form the [Mets7+Pt]^2+^ species, the structuring effect induced by the Pt(II) ion (devoid of all its original ligands) is even more evident. Conversely, when Mets7 reacted with oxaliplatin, the effect of metal binding to the peptide is moderate, as evidenced by the reduction of the band at 195 nm, indicating only a slight conformational change of the peptide (Figure 4B). Hence, the CD data reflect the different reactivity of kiteplatin and oxaliplatin toward Mets7: oxaliplatin, which retains the 1*R*,2*R*‐DACH ligand, causes a moderate conformational change, whereas kiteplatin, whose ligands are completely replaced by Mets7 donor atoms, influences the peptide conformation more deeply (Figure 4). It is plausible that the methionine residues of the peptide bind to kiteplatin as in the case of cisplatin,^[28]^ causing translabilization of both ancillary and leaving ligands.

**Figure 5:**
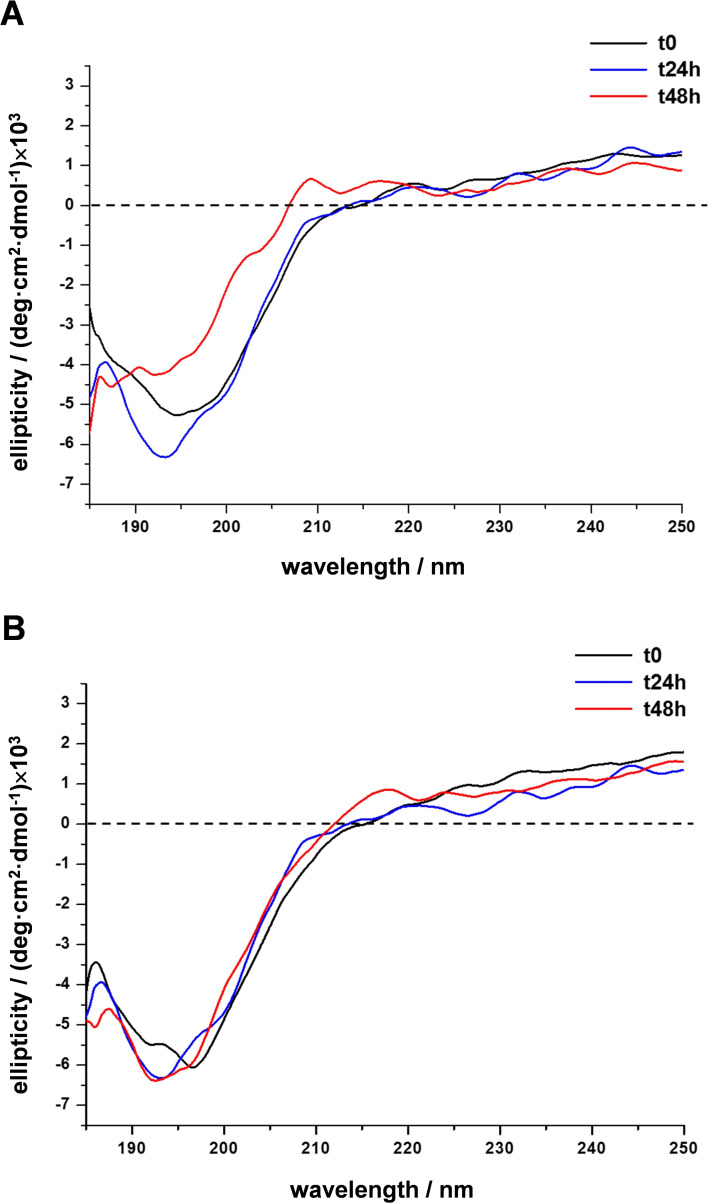
Far‐UV circular dichroism spectra of Mets7 incubated with one equivalent of [Pt(*cis*‐1,4‐DACH)(oxa)] (**3**) (A) and [Pt(*cis*‐1,4‐DACH)(cbdca)] (**4**) (B) recorded at different time intervals.

After addition of one equivalent of **3** to Mets7 no changes in the CD spectrum were observed up to 24 h (Figure 5A). After 48 h of incubation, the band at 195 nm decreased slightly. It is deduced that the effect of complex **3** on the peptide structure is similar to that observed with oxaliplatin. On the contrary, no structural modifications of the peptide were observed upon reaction with complex **4**; in fact, the CD profile remained unchanged after 48 h (Figure 5B).

**Figure 6:**
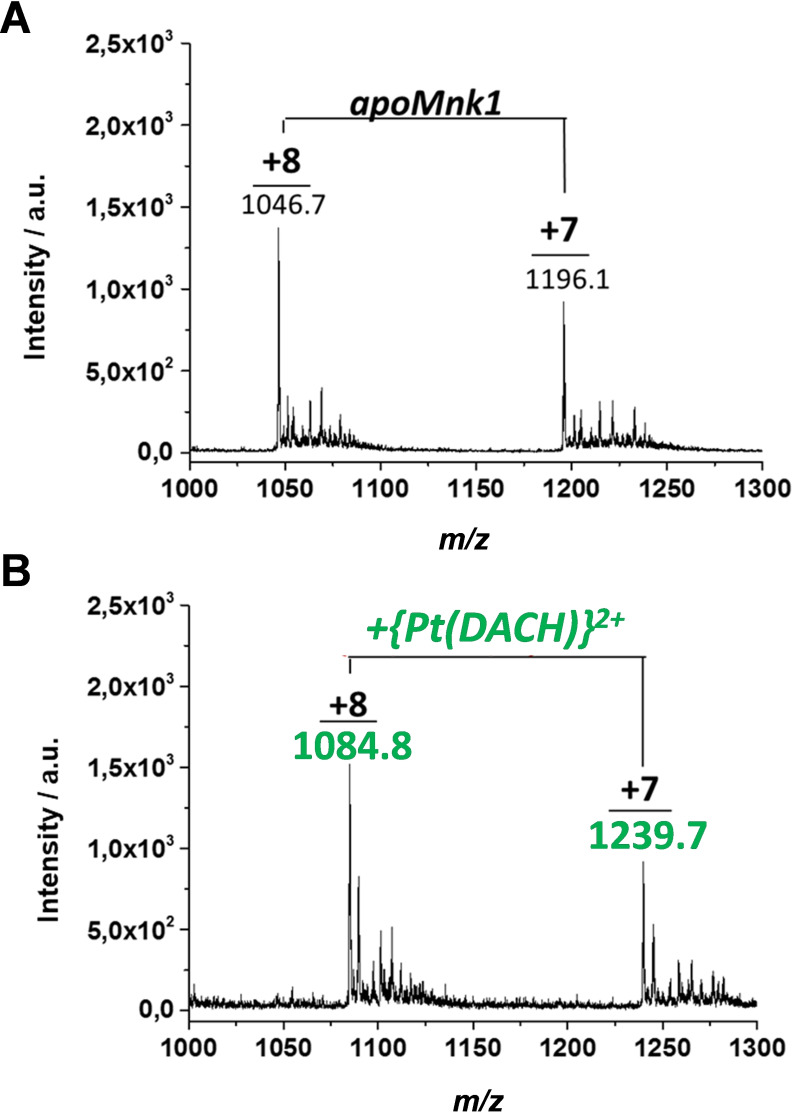
ESI‐MS spectra of Mnk1 incubated with an equimolar amount of kiteplatin (**1**), recorded at time zero (A) and after 24 h of incubation (B).

The reactivity of kiteplatin and oxaliplatin toward Mnk1 was also investigated. Mnk1 is the first N‐terminal cytoplasmic domain of ATP7A consisting of 73 residues and characterized by a surface‐exposed Cu(I)‐binding site composed of two closely spaced cysteines. The CxxC motif is also the preferred binding site of Pt(II) drugs. Mnk1 was shown to coordinate to cisplatin and oxaliplatin through Cys residues, with the Pt ion retaining the ammine ligands.^[26]^ To investigate the reactivity of kiteplatin with Mnk1, the Pt complex was added to the protein in equimolar amount (100 μM) under anaerobic conditions, and the reaction was monitored by ESI‐MS (see Table 3). Soon after mixing, the ESI‐MS spectrum showed two peaks at *m/z* 1046.7 (8+ charge) and 1196.1 (7+ charge), corresponding to the *apo*protein (Figure 6A). After 24 h of incubation, two peaks at *m/z* 1084.8 (8+ charge) and 1239.7 (7+ charge) appeared, corresponding to the adduct with the {Pt(DACH)}^2+^ moiety (Figure 6B), similar to that observed when the protein reacted with oxaliplatin.

**Table 3:** Assignment of adducts of Mnk1 incubated with kiteplatin (**1**) and [Pt(*cis*‐1,4‐DACH)(oxa)] (**3**) observed by ESI‐MS.

Adduct assignment	Experimental *m/z* ^[a]^	Calculated *m/z* ^[a]^
*apo*Mnk1		
[Mnk1+7H]^7+^	1196.1	1196.0
[Mnk1+8H]^8+^	1046.7	1046.7
Mnk1+**1**		
[Mnk1+Pt(1,4‐DACH)+5H]^7+^	1239.7	1239.9
[Mnk1+Pt(1,4‐DACH)+6H]^8+^	1084.8	1085.0
Mnk1+**3**		
[Mnk1+Pt(1,4‐DACH) (oxa)+7H]^7+^	1252.6	1252.8
[Mnk1+Pt(1,4‐DACH)+5H]^7+^	1239.7	1239.9

[a] The reported *m/z* ratios are referred to the prevailing isotopologue in the isotope pattern.

**Figure 7:**
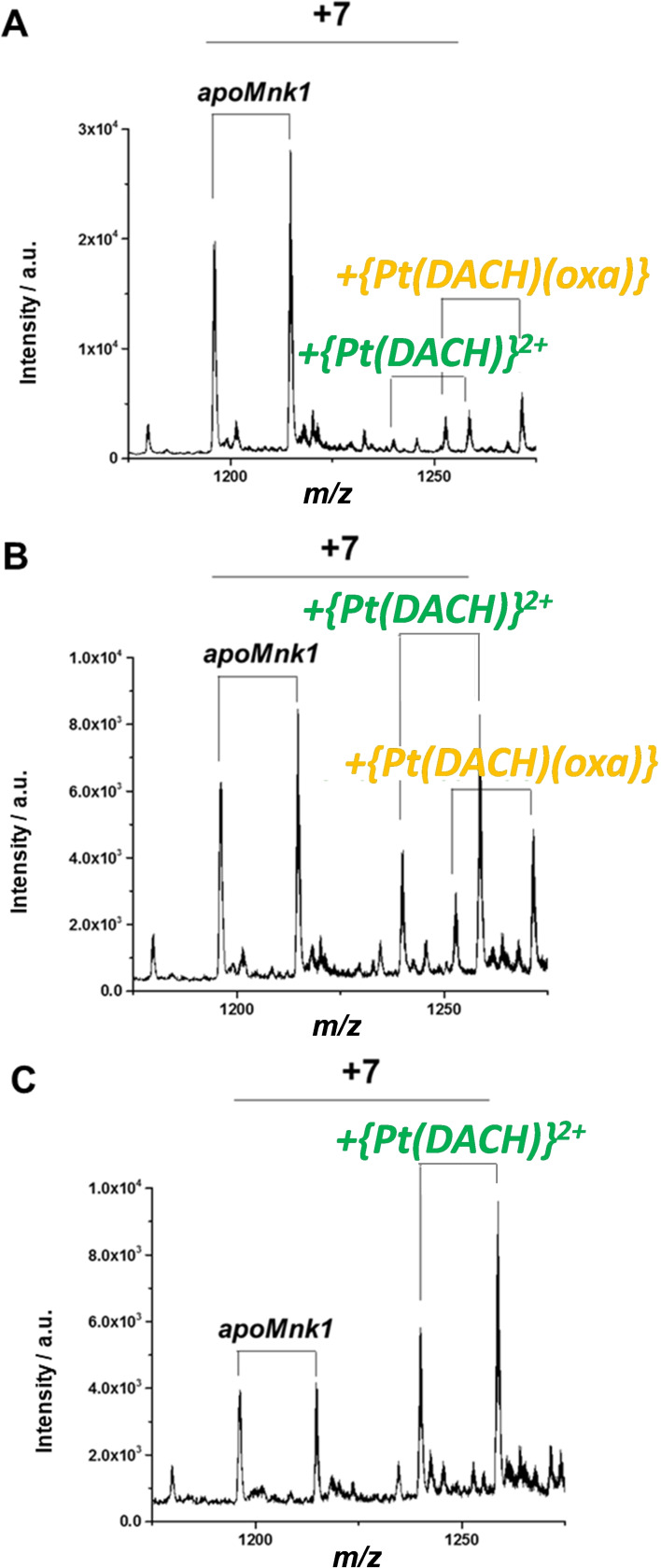
ESI‐MS spectra of Mnk1 incubated with an equimolar amount of [Pt(*cis*‐1,4‐DACH)(oxa)] (**3**), recorded at time zero and after 24 h and 48 h of incubation. Each species comprises two pairs of signals corresponding to Mnk1 with and without the first methionine residue.

With the two derivatives of kiteplatin (complexes **3** and **4**), the reactions were carried out under the same conditions described above. Sample aliquots were 3‐fold diluted and analyzed by ESI‐MS at time zero and after 24 h and 48 h incubation. The ESI‐MS spectra recorded in the reaction with complex **3** (having the oxalate leaving group) are reported in Figure 7. A barely detectable amount of Pt‐protein adducts was present at time zero. After 24 h of reaction, the peaks at *m/z*=1252.6 and 1239.7 (7+ charge), corresponding to mono‐ and bidentate adducts of Mnk1 with {Pt(DACH)(oxa)} and {Pt(DACH)}^2+^ respectively, increased in intensity. After 48 h of reaction, the peaks corresponding to the monodentate adduct (i. e. Mnk1 binding to {Pt(DACH)(oxa)} through only one Cys) were no longer detectable due to the loss of the oxalate group, but a residual amount of *apo*Mnk1 was still present.

No binding of Pt to the protein was observed when Mnk1 was reacted with complex **4**, which retained the cbdca leaving group for up to 48 h of reaction (data not shown).

Since the interaction of Pt‐based drugs with copper transport proteins deeply influences their cellular accumulation and hence their activity, the present investigation is a first step in the understanding of the different ability of complexes **1**, **3**, and **4** to overcome cisplatin resistance. Further studies concerning the reactivity toward intracellular nucleophiles or the involvement of other transporters, such as OCTs, are necessary to elucidate the different mechanism of action that makes kiteplatin so different from its derivatives.

## Conclusion

In the present work, using the ESI‐MS and CD spectra, we demonstrated that when the Mets7 peptide is incubated with kiteplatin, the latter loses the carrier ligand *cis*‐1,4‐DACH as well as one or two chlorido ligands, and the Pt(II) ion binds to the peptide as occurs with cisplatin. The oxalate derivative **3** exhibits the same behavior, albeit with a slower reaction rate. On the other hand, oxaliplatin retains the 1*R*,2*R*‐DACH ligand after 48 h of reaction. Oxaliplatin and complex **3** have the same molecular formula but a different spatial position of the −NH_2_ groups on the DACH carrier ligand. This change could explain the different reactivity of **3** and oxaliplatin toward Mets7. The 1*R*,2*R*‐DACH ligand forms a stable 5‐membered chelate ring with Pt(II), while 1,4‐DACH forms a loose 7‐membered ring. The least reactive compound is complex **4**; in the ESI‐MS spectrum recorded after 48 h no peak corresponding to an adduct with the peptide was observed, probably due to the steric hindrance of the cbdca leaving group.

Kiteplatin and oxaliplatin have similar reactivity toward the first domain of ATP7A, as they maintain the DACH group and lose the chlorido and oxa ligands, respectively. The effect of the leaving group is evident in the reaction of Mnk1 with **3** and **4**: while the oxalate group in complex **3** is displaced by Mnk1 (which binds the {Pt(*cis*‐1,4‐DACH)}^2+^ moiety), the cbdca ligand in complex **4** hinders the coordination of the protein.

## Experimental Section

***Synthesis of Pt complexes***. [PtCl_2_(*cis*‐1,4‐DACH)] (kiteplatin, **1**),^[6]^ [Pt(1*R*,2*R*‐DACH)(oxa)] (oxaliplatin, **2**),^[39]^ and [Pt(*cis*‐1,4‐DACH)(cbdca)] (**4**)^[6]^ were prepared as reported in the literature.

[Pt(*cis*‐1,4‐DACH)(oxa)] (**3**): oxalic acid [(COOH)_2_, 0.041 g, 0.45 mmol] was dissolved in water (40 mL) and the resulting solution was treated in the dark with Ag_2_O (0.104 g, 0.45 mmol) for 15 min at 50 °C. The resulting suspension was added to **1** (0.171 g, 0.45 mmol), stirred with a magnetic stirrer in the dark at 50 °C for 90 min and then at room temperature for 24 h. The white precipitate that formed (AgCl) was filtered through celite and the filtrate evaporated under vacuum at 40 °C. The light yellow residue (0.134 g, 0.34 mmol; yield 75 %) corresponds to [Pt(*cis*‐1,4‐DACH)(oxa)].

^1^H NMR: (D_2_O) 5.29 (4H, NH_2_), 3.21 (2H, CH), 1.76 (8H, CH_2_) ppm. ^195^Pt NMR: (D_2_O) −1865 ppm. ESI‐MS: *calculated for* [C_8_H_14_N_2_O_4_PtNa]^+^ [**3**+Na]^+^: 420.0. *Found*: *m/z* 420.0. Anal.: *calculated for* C_8_H_16_N_2_O_5_Pt (**3** ⋅ H_2_O): C, 23.14; H, 3.88; N, 6.74 %. *Found*: C, 23.44; H, 3.61; N, 6.73 %.

***Cytotoxicity assays***. The number of living cells was evaluated by the 3‐(4,5‐dimethylthiazol‐2‐yl)‐2,5‐diphenyltetrazolium bromide (MTT) assay. Briefly, A2780 human ovarian endometrioid adenocarcinoma cells and cisplatin‐resistant A2780/Cp8 ovarian cancer cells were plated in 96‐well plates at a density of ∼5000 cells/well. After one day incubation at 37 °C in a humid atmosphere with 5 % CO_2_, the culture medium was replaced with 100 μL of fresh medium (control cells) or medium containing different concentrations of the test compounds in the range 0.125–50 μM. Untreated cells were used as positive control. After the incubation period of 72 h, 10 μL of a 0.5 % (*w/v*) MTT/PBS solution were added to each well and the incubation was prolonged further for 3 h. After this period, medium was removed and replaced with 100 μL of DMSO. The absorbance of the individual well was measured by a microplate reader (MULTISKAN Sky, ThermoScientific). Each compound concentration was tested in triplicate, and results presented as percentage of the control value. IC_50_ values were calculated using nonlinear regression in GraphPad Prism 5.01.

***Sample preparation for ESI‐MS experiments***. The Pt complexes were dissolved immediately prior to use in pure water at 1 mM final concentration. The complex solution was extensively vortexed and sonicated. The peptide Mets7, acetylated at its N‐terminus, was purchased from GenScript Corp. (USA). The purity was validated to be greater than 98 % by analytical HPLC and the mass was confirmed by ESI‐MS (calculated MW: 883.4; found 883.5). Mets7 was dissolved in pure water at 1 mM concentration. Mnk1 was recombinantly expressed by standard biotechnological techniques, using *Escherichia coli* BL21‐Gold (DE3) competent cells (Stratagene, US) and pET vectors under the control of the Isopropyl‐β‐D‐1‐thiogalattopyranoside (IPTG) inducible *lac* promoter (Novagen, US). Purification was achieved by immobilized metal affinity and size‐exclusion chromatography on an automated AKTA‐Purifier UPC900, equipped with a detector at 280 nm. After cleavage of the (His)_6_ purification tag, four amino acids (IEGR) of the restriction site were left at the C‐terminus of the protein. The N‐terminal methionine was partially processed in *E. coli*, yielding a 76‐amino acid protein (numbering the first codon Met as residue 1). All purification steps were carried out in the presence of an excess of the reducing agent dithiothreitol (DTT), to preserve the protein in its active form. Protein purity was determined by SDS‐PAGE and ESI‐MS (calculated MW: 8365.5; found: 8365.6). To extensively remove DTT prior to Pt‐drug addition, protein samples were washed with deoxygenated water by using Amicon Ultracentrifugal filters with 3 kDa cutoff (Millipore, US), under N_2_ atmosphere, and immediately used for the incubations.

***Electrospray ionization mass spectrometry***. ESI‐MS was performed with an electrospray interface and an ion trap mass spectrometer (1100 Series LC/MSD Trap system, Agilent, USA).

The reaction between Mets7 (50 μM) and kiteplatin (50 μM) or oxaliplatin (50 μM) was carried out at 25 °C. Aliquots of the reaction mixture were removed at different time intervals after mixing and injected at a rate of 10 μL/min. 1 % acetic acid (*v/v*) was added before injection in order to obtain a good volatilization. Ionization was achieved in the positive ion mode by application of +4 kV at the entrance of the capillary; the pressure of the nebulizer gas was 15 psi. The drying gas was heated to 350 °C at a flow rate of 5 L/min. Full‐scan mass spectra were recorded in the mass/charge (*m/z*) range of 50–2200. In the reaction with Mnk1, an equimolar amount of **1**, **3** or **4** was added to Mnk1 (100 μM) at 25 °C under anaerobic conditions. Aliquots were removed at different time intervals, 3‐fold diluted, and analyzed by ESI‐MS as previously described.

***Inductively coupled plasma mass spectrometry***. The Pt uptake in A2780 and A2780/Cp8 cells was evaluated by ICP‐MS using an iCAP Q mass spectrometer (Thermo Fisher Scientific, MA, USA). The cells were seeded in 60 mm tissue culture dishes at a density of 500,000 cells and incubated at 37 °C in a humidified atmosphere with 5 % CO_2_. After overnight incubation, the culture medium was replaced with 3 mL of medium containing the compounds at 10 μM concentration and incubated for 4 h and 24 h. After the incubation period, the cell monolayer was washed twice with ice‐cold PBS. The samples were vortexed in the presence of 500 μL of 65 % HNO_3_ (Merck, Germany) and then digested overnight at room temperature. Finally, the solutions were diluted to 10 mL with Milli‐Q water. Tune A solution (Thermo Fisher Scientific, MA, USA) was used to tune the ICP‐MS instrument. Bismuth was used as an internal standard, hence 20 μL of its solution at 500 μg/L were added to the samples prior to analysis. All samples were analyzed three times. The isotopes monitored were ^195^Pt and ^209^Bi and the Pt concentration was determined by comparison with an external calibration curve (5 concentration levels in the interval 0.1–5.0 μg/L). The detection limit was calculated as the concentration of an element that gave a signal equal to three times the standard deviation of a series of ten successive measurements of the blank solution.

***Circular dichroism***. Far‐UV CD spectra were recorded at different time intervals on *apo*Mets7 (50 μM) incubated with **1‐4** (50 μM) in pure water using a Jasco J‐810 spectropolarimeter (Jasco Inc., Easton, MD, USA), a quartz cuvette with a 1 mm optical path, a wavelength interval of 190–250 nm and 0.1 nm data pitch. All spectra, corresponding to an average of 10 scans, were baseline corrected and then smoothed by applying adjacent averaging or a fast Fourier transform filter. The ellipticity is reported as mean residue molar ellipticity (deg ⋅ cm^2^ ⋅ dmol^−1^) according to [θ]=100 [θ]_obs_/(C ⋅ L ⋅ N), where [θ]_obs_ is the observed ellipticity in degrees, C is the molar concentration of the protein, L is the optical path length (in cm), and N is the number of amino acids (N=8 for Mets7).

## Conflict of interest

The authors declare no conflict of interest.
